# Autophagy in *Staphylococcus aureus* Infection

**DOI:** 10.3389/fcimb.2021.750222

**Published:** 2021-10-07

**Authors:** Mengyao Wang, Ziyao Fan, Hongbing Han

**Affiliations:** ^1^ Beijing Key Laboratory of Animal Genetic Improvement, College of Animal Science and Technology, China Agricultural University, Beijing, China; ^2^ National Engineering Laboratory for Animal Breeding, College of Animal Science and Technology, China Agricultural University, Beijing, China; ^3^ Key Laboratory of Animal Genetics, Breeding and Reproduction of the Ministry of Agriculture and Rural Affairs, College of Animal Science and Technology, China Agricultural University, Beijing, China; ^4^ Genome Analysis Laboratory of the Ministry of Agriculture and Rural Affairs, Agricultural Genomics Institute at Shenzhen, Chinese Academy of Agricultural Sciences, Shenzhen, China

**Keywords:** *Staphylococcus aureu*s, autophagy, accessory gene regulatory system, intracellular persistence, host-pathogen interactions

## Abstract

*Staphylococcus aureus* is an invasive, facultative intracellular pathogen that can colonize niches in various host organisms, making it difficult for the host immune system to completely eliminate. Host autophagy is an intracellular clearance pathway involved in degrading *S. aureus*. Whereas the accessory gene regulatory system of *S. aureus* that controls virulence factors could resist the host immune defenses by evading and even utilizing autophagy. This article reviews the interaction between autophagy and *S. aureus*, providing insights on how to use these mechanisms to improve *S. aureus* infection control.

## Introduction

*Staphylococcus aureus* is an opportunistic pathogen that has adapted to long-term colonization in the human skin and nares (Jeon et al., 2020). *S. aureus* utilizes the adhesins to initiate the invasion process by attaching to the surface of host cell (Horn et al., 2018; Watkins and Unnikrishnan, 2020). After invasion, *S. aureus* induces a cytoplasmic and mitochondrial Ca^2+^ overload, which leads to both apoptotic and necrotic cell death (Stelzner et al., 2020). *S. aureus* infection presents as long-lasting persistent or acute diseases that are associated with significant morbidity and mortality (Turner et al., 2019). Antibiotics were most widely used to treat *S. aureus* infectious diseases, however, *S. aureus* has rapidly developed resistance to antibiotics. Approximately 90% of *S. aureus* strains show resistance to multiple antibiotics, resulting in decreased antibiotic application and reduced antibiotic effectiveness (Costa et al., 2018). Since methicillin-resistant *S. aureus* (MRSA) was identified in 1960, the infection rate with MRSA has increased globally. It leads to an increased burden on healthcare-associated expenditures (Zhen et al., 2020), and has become the main cause of bacterial infection in hospitals and communities (Lakhundi and Zhang, 2018). MRSA strains account for 5%-82% of *S. aureus* isolates (Köck et al., 2010; Falagas et al., 2013), leading to clinical syndromes including bacteremia (Klevens et al., 2007), one of the most severe situations of *S. aureus* infections with 15%-60% mortality rates (Li et al., 2021). Invasive MRSA strains possess a series of virulence factors and toxins, allowing them to spread rapidly in the community, and seriously threaten public health (Lakhundi et al., 2018). Therefore, new strategies to control *S. aureus* infection have gradually become the focus by manipulating and enhancing host immune defenses (Keller et al., 2020; Gauron et al., 2021).

The host immune system provides the first defense against pathogens, effectively removing intracellular pathogens in most cases. Simultaneously, autophagy also plays an essential role in resisting to pathogens (Randow et al., 2013). Autophagy is a fundamental biological process, in which pathogens are engulfed by double membrane vesicles called phagophores and eventually transported to lysosomes for subsequent degradation (Kirkegaard et al., 2004; Nakatogawa, 2020). Recent studies have demonstrated that autophagy has a crucial role in host cell defense against *S. aureus* (Lv et al., 2019; Gibson et al., 2020). The cell wall components of *S. aureus* can be detected as pathogen-associated molecular patterns (PAMPs) and then induce autophagy (Arroyo et al., 2013; Wu et al., 2016). Autophagy effectively limits *S. aureus* growth by fusion with the lysosome or positively regulating the phagocytosis of macrophages (Lv et al., 2019; Gibson et al., 2020). Researchers have thus tried to use the autophagy pathway to control *S. aureus* infection. Whereas some *S. aureus* strains have evolved self-defense mechanisms against autophagy degradation, and are even protected by the autophagy pathway (Schnaith et al., 2007). Once *S. aureus* enters the autophagosome, it transforms this “compartment” to create a hospitable environment in which it can survive and replicate (O'Keeffe et al., 2015). *S. aureus* being degraded by the autophagy pathway or protected by the autophagosome compartment is related to the accessory gene regulatory (*agr*) system which plays a crucial role in pathogenesis by coordinating virulence factors expression and bacterial density (Schnaith et al., 2007; O'Keeffe et al., 2015).

Here, we reviewed “beneficial” and “harmful” functions of autophagy in the process of *S. aureus* infection, as well as the mechanism by which *S. aureus* evades autophagy. This review is helpful to understand the interaction between hosts and *S. aureus*, and provides a theoretical basis for the development of new treatments for *S. aureus* infection.

## *S. aureus* can Infect Host as a Facultative Intracellular Pathogen

Based on phylogenetic analyses, Queck et al. reported that *S. aureus* first emerged as a nonvirulent species, and only later acquired virulent functions (Queck et al., 2008). The *agr* quorum sensing system is the main virulence regulator of *S. aureus* in response to changing environmental conditions, such as adapting to low-nutrition conditions in high-cell-density populations, forming a nonpathogenic lifestyle (Queck et al., 2008). Approximately 30% of humans persistently but asymptomatically carry *S. aureus* in their nasopharynx (Wertheim et al., 2005). *S. aureus* actively adheres to promote colonization and replicates to avoid removal by nasal secretions (Foster et al., 2014).

The cell wall-anchored proteins of *S. aureus*, Fnbps and IsdB, promote internalization and subsequent invasion (Zapotoczna et al., 2013; Schlesier et al., 2020). The pattern recognition receptors (PRRs) expressed on the surface of phagocytes recognize pathogens and mediate their uptake into phagosomes for later elimination (Flannagan et al., 2009). Nonprofessional phagocytes utilize endocytosis to take up *S. aureus* (Moldovan and Fraunholz, 2019). Once internalized by host cells, the *agr* system of *S. aureus* increases virulence factors to damage phagosomes and promote intracellular survival (Novick et al., 1993). The phagosome or endosome can fuse directly with a lysosome to acidify to low pH for degrading microorganisms (Flannagan et al., 2009; Lâm et al., 2010). However, *S. aureus* tolerates acidic environments, which contributes to its survival within phagolysosomes (Weinrick et al., 2004). Exposure to an acidic environment increased expression of *agr* system (Tranchemontagne et al., 2016). Phagosomal acidification even appears to be essential for survival of some *S. aureus* strains (Tranchemontagne et al., 2016). *Agr* positively regulates cytotoxic phenol-soluble modulins (PSMs), which mediate escape from the phagosome into the cytoplasm to avoid lysosomal killing (Grosz et al., 2014; Münzenmayer et al., 2016). The cytoplasmically located *S. aureus* or leaky phagosomes could be captured by autophagosomal membranes and eventually fuse with lysosomes for autophagic degradation (Fraunholz and Sinha, 2012). *S. aureus* is also capable of escaping or even manipulating the autophagy pathway for replication and dissemination (Vozza et al., 2021). *S. aureus* further evolved regulatory functions to attenuate the expression of virulence genes to reduce innate immune defenses (Boisset et al., 2007; Cheung et al., 2014). This decreases the pro-inflammatory potential of *S. aureus*, which is associated with chronic infection. Surprisingly, *S. aureus* is very responsive to external stimuli, and rapidly reverts back to the original virulent state in rich bacterial growth conditions (Tuchscherr et al., 2020).

## The Effect of Autophagy on Intracellular *S. aureus*

**Figure 1:**
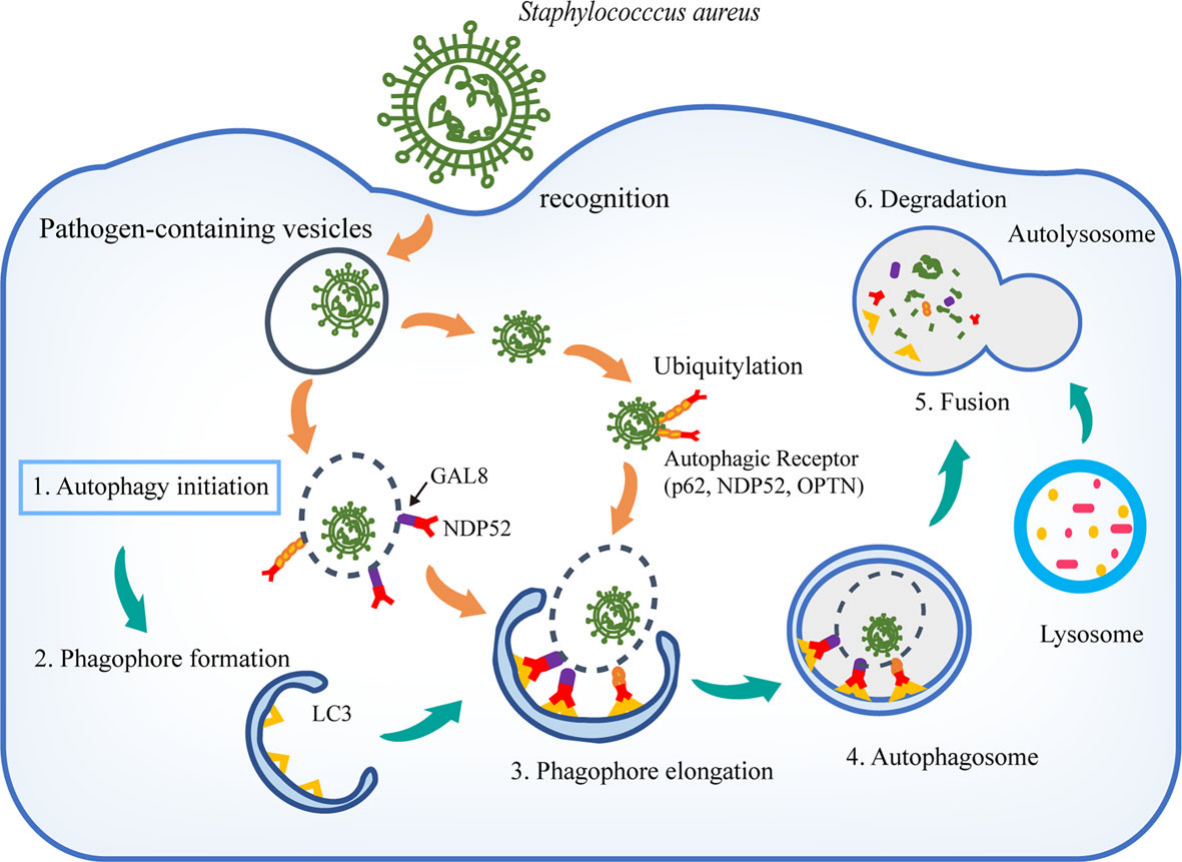
Host autophagy in defense against *S. aureus*. The components of *S. aureus* are detected as PAMPs by host PRRs, and autophagy is induced. Autophagy receptors p62, NDP52, and OPTN function as bridging adaptors to induce selective autophagic degradation of invading pathogens by specifically recognizing ubiquitin-coated intracellular pathogens. Damaged pathogen-containing vesicles are detected by GAL8. GAL8 monitors endosomal integrity and activates antibacterial autophagy in conjunction with the autophagy receptor NDP52. During autophagy, LC3 is recruited to autophagosomal membranes. Autophagosome subsequently fuses with a lysosome to form the autolysosome, where the acidic environment and enzymes mediate the bacterial degradation.

Autophagy is considered a crucial intracellular degradation system for removing dangerous pathogens (Levine, 2005). The dynamic membrane processes of autophagy occur through regulators comprised of autophagy-related genes (ATGs) and additional factors based on the following sequential steps: autophagy initiation; phagophore formation; double-membrane nucleation and phagophore elongation; cytoplasmic microorganism engulfment; autophagosome fusion with lysosome; and cargo degradation (Kuo et al., 2018) (**Figure 1**).

### Manipulation of Autophagy on *S. aureus* in Non-Professional Phagocytes

In the non-professional phagocytes, intracellular *S. aureus* is rapidly ubiquitinated and then recognized by autophagy receptors, including sequestosome 1 (SQSTM1/p62), nuclear domain protein 52 (NDP52/CALCOCO2), and optineurin (OPTN) (Neumann et al., 2016) (**Figure 1**). These receptors characteristically couple ubiquitin to microbes with the autophagosomal membrane-associated protein LC3, to trap bacteria in autophagosomes (Mestre et al., 2010; Neumann et al., 2016). Endosomes that are damaged by *S. aureus* are targeted by Galectin-8 (GAL8) to attract autophagosomal clearance (Soong et al., 2015). Phospholipase C-related catalytically inactive protein (PRIP) has been shown to be required for the autophagosome maturation and acidification, which facilitates the *S. aureus* elimination by promoting the fusion of *S. aureus*-containing autophagosomes with lysosomes in mouse embryonic fibroblasts (Harada-Hada et al., 2014). Recently, the positive role of autophagy was further supported by autophagy protein which mediates a novel form of defense in response to *S. aureus* infection. ATG16L1 protects host cells from *S. aureus* by releasing ADAM10 (a disintegrin and metalloproteinase 10) as a bacterial toxin scavenger in alveolar epithelial cells. Loss of ATG16L1 expression exacerbates *S. aureus*-induced mortality in mice (Becker et al., 2014; Keller et al., 2020). Except for the above resistance mechanisms that reduce *S. aureus* burden, autophagy could protect host cells against *S. aureus* infection by maintaining tolerance toward the pore forming alpha-toxin (α-toxin) secreted by *S. aureus* (Maurer et al., 2015). Increased cell death induced by α-toxin was observed in mouse endothelial cells upon autophagy inhibition, revealing that autophagy was a barrier of cells to maintain membrane homeostasis under stress conditions (Maurer et al., 2015).

However, *S. aureus* has developed mechanisms to escape from the autophagy pathway (Riebisch et al., 2021). It has been demonstrated that *S. aureus* can block autophagosome maturation *via* phosphorylation of mitogen-activated protein kinase 14 (MAPK14) and ATG5 in murine fibroblasts (Neumann et al., 2016). *S. aureus* secreted α-toxin was shown to inhibit the fusion of autophagosomes with lysosomes to prevent *S. aureus* degradation before reaching the cytoplasm (Mestre et al., 2010). The *S. aureus*-containing autophagosomes neither acidified nor acquired lysosome-associated membrane protein-2 (LAMP-2), a marker for late endosomes and lysosomes. This dysfunctional autophagic response was also observed in *S. aureus* infected bovine mammary epithelial cells (Wang et al., 2019). After escape from autophagosomes, *S. aureus* proliferates extensively in the cytoplasm and eventually results in the lysis of host cell (Schnaith et al., 2007). In addition to avoiding autophagy, some *S. aureus* have developed to utilize autophagy for their own benefit in host cells. Na Geng et al. described that *S. aureus* caused obvious induction of autophagosomes formation to facilitate intracellular replication in bovine mammary epithelial cells (Geng et al., 2020). It was also supported by a recent study that autophagy suppressed by overexpression of protein kinase C (PKC) could inhibit *S. aureus* intracellular replication in Chinese hamster ovary cells (Gauron et al., 2021). Additionally, Bravo-Santano et al. demonstrated glucose and amino acid pools were severely depleted by *S. aureus* to induce a starvation response, which leads to highly activated glutamine in host cells for their own metabolic needs. These changes activate autophagy through AMP-activated protein kinase (AMPK) and extracellular signal-regulated kinase (ERK) signaling pathways. Metabolic activation of autophagy is used by *S. aureus* to sustain its own intracellular survival (Bravo-Santano et al., 2018).

### The Effect of Autophagy in *S. aureus* Infected Professional Phagocytes

In professional phagocytes, phagocytosed *S. aureus* are initially located in a phagocytic vesicle. The vacuolar pathogens can be sequestered into autophagic membranes to thereby eventually fuse with lysosomes (Knodler and Celli, 2011). The autophagy receptor SQSTM1/p62 has been shown to directly co-localize with *S. aureus* in the cytosol in neutrophils for autophagic degradation. SQSTM1/p62 knockdown significantly impaired host defense and increased susceptibility of neutrophils to *S. aureus* (Gibson et al., 2020). Besides being an autophagy receptor, SQSTM1/p62 brings the precursor protein of ribosomal protein S30 and additional ubiquitinated protein complexes to autolysosomes, where they were processed from innocuous forms into bactericidal products (Ponpuak et al., 2010). Thus, SQSTM1/p62 is crucial in antibacterial action in host cells. Autophagy also controls *S. aureus* infection by promoting phagocytosis in macrophages. Decreased level of autophagy through the PI3K inhibitor LY294002 or knockdown of Beclin1 treatment significantly weakens phagocytosis of *S. aureus*-infected macrophages, indicating that *S. aureus*-induced autophagy contributes to the phagocytosis of macrophages (Lv et al., 2019). Moreover, the intracellular autophagy-related molecule microtubule-associated protein 1S (MAP1S) promotes phagocytosis of *S. aureus* by enhancing the MyD88-dependent TLR signaling pathway. The Map1S-deficient macrophages exhibit impaired *S. aureus* phagocytosis (Shi et al., 2016). These lines of evidence demonstrate autophagy has a crucial role in eliminating *S. aureus*.

By contrast, this cellular defense program has also been identified as providing a niche for intracellular *S. aureus* replication. Some studies reported *S. aureus* are protected from degradation within autophagosomes of phagocytes, and have obtained an intracellular survival niche, which ultimately facilitates dissemination in the host (O'Keeffe et al., 2015; Mulcahy et al., 2020). *S. aureus* escapes autophagic degradation by blocking autophagy flux (LC3-II, p62) and increasing the pH in autolysosomes after invading macrophages (Cai et al., 2020). It has been reported chemical inhibition of the autophagic response by 3-methyladenine (3-MA) promoted phagocytosis of mouse macrophages (Zhu et al., 2018) and prevented the escape of *S. aureus* in mouse bone marrow-derived dendritic cells (O'Keeffe et al., 2015). These data indicate that inhibiting the formation of autophagosomes facilitates elimination intracellular *S. aureus*. *S. aureus* also have developed to utilize autophagy in professional phagocytes. In primary human polymorphonuclear neutrophils (PMNs), *S. aureus* enhances the accumulation of autophagosomes in cells by activating the stress response pathway to maintain the survival niche (Mulcahy et al., 2020). At the meantime, *S. aureus* could disrupt the apoptotic pathway of PMNs to prevent the destruction of its intracellular niche and protect itself from subsequent macrophages phagocytosis (Vozza et al., 2021). The non-canonical form of autophagy machinery LC3-associated phagocytosis (LAP), which is dependent on NADPH oxidase, can also be utilized by intracellular *S. aureus* for pathogenesis. At the early stage of infection in zebrafish neutrophils, the autophagy marker LC3 rapidly decorates *S. aureus*-containing single-membrane phagosomes. The formation of LC3-positive and non-acidified phagosomes provide a spacious area for *S. aureus* to safely replicate (Prajsnar et al., 2020).

## The Effect of *Agr* on Autophagy Controlling Intracellular *S. aureus*

*Agr* system is a major gene regulator that governs the toxin production of *S. aureus* at the appropriate time, regulating the adhesins expression during attachment and virulence factors during infection. *Agr* can upregulate α-toxin to cause tissue destruction by perturbing to epithelial cell junctions (von Hoven and Husmann, 2019). α-toxin also increased *S. aureus* internalization within mast cells by up-regulation of β1 integrin (Goldmann et al., 2016). After internalization, the high-level expression of *agr* led to strong expression of toxins and exoenzymes, as well as increased expression of methicillin resistance genes, mediating the pathogenesis (Cheung et al., 2011). At the meantime, the *agr* locus controlled phenol-soluble modulins alpha (PSMα) has also been shown to be crucial for phagosomal escape in both professional and non-professional phagocytes (Grosz et al., 2014). When *agr* is absent, phagosomal escape and autophagosomal accumulation are significantly reduced as well as intracellular bacterial burden is reduced (O'Keeffe et al., 2015; Blättner et al., 2016). Additionally, *agr* has been shown to have the alternating function, which can reduce cytotoxicity to survive persistently within host cells and avoid the host immune system activation (Tuchscherr et al., 2011).

**Figure 2:**
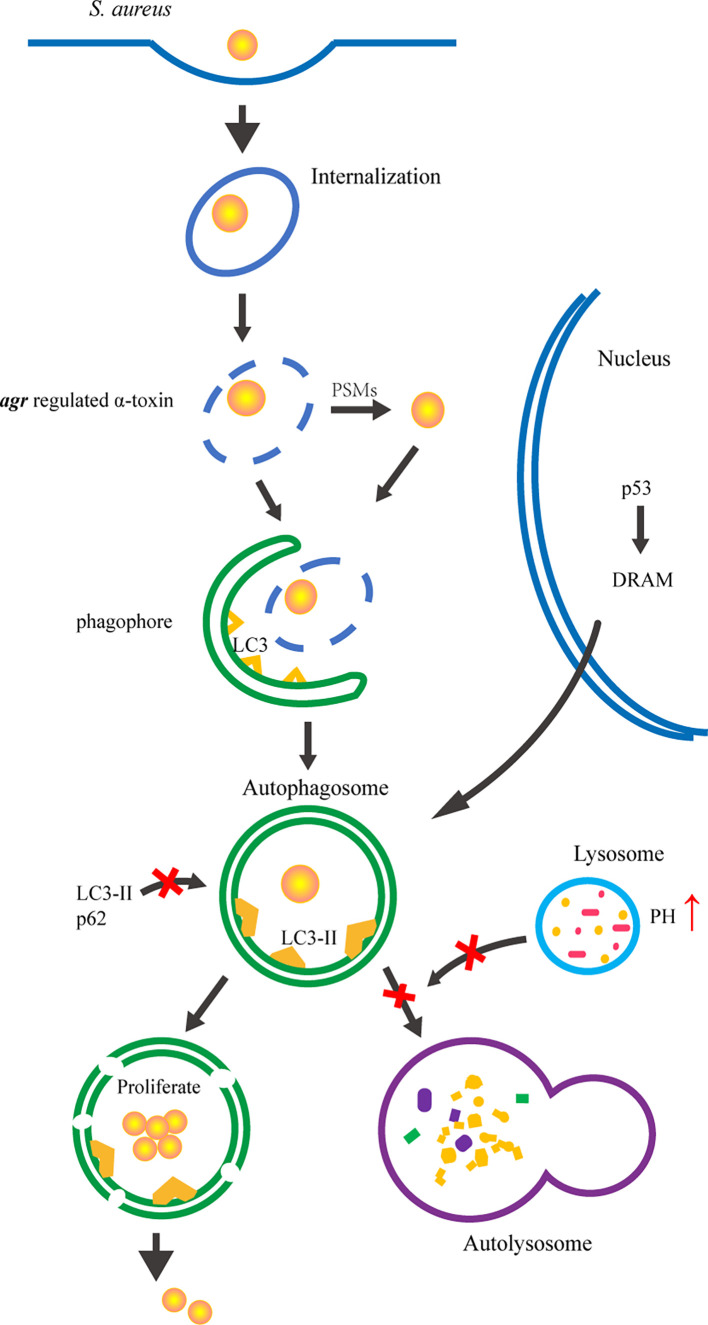
The interaction between autophagy and *agr* locus of *S. aureus*. *S. aureus* is internalized into host cell *via* the endocytic pathway. *Agr* system positively regulates α-toxin and PSMs to mediate escape from the phagosome into cytoplasm. The escaped *S. aureus* and damaged phagosomes are engulfed by phagophores. The expression of LC3-II and p62 are blocked by *agr* system to hinder autophagosomal maturation. Upon autophagosomal maturation, *agr* as the pH sensitive system inhibit the fusion of autophagosome and lysosome to escape autophagic degradation. Thus, the autophagosomes provide a niche for *S. aureus* replication. In addition, *agr*-specific factor was found to accumulate autophagosomes as intracellular survival niches by manipulating the p53/DRAM pathway in human PMNs, whereas has not been found in other species or cell types. Eventually, *S. aureus* escape from autophagosomes into the cytoplasm and induces host cell death.

Schnaith et al. reported *agr*-regulated factor(s) activated autophagy could prevent the maturation of *S. aureus-*containing phagosomes in human epithelial cells (Schnaith et al., 2007). Subsequently, the *agr* regulated α-toxin was shown to be necessary for eliciting autophagy, but the autophagic response was dysfunctional and the induced autophagosomes were not acidic. Additionally, α-toxin-deficient *S. aureus* strains were unable to activate the autophagy pathway (Mestre et al., 2010) (**Figure 2**). In addition, an *agr*-specific factor was discovered that manipulates the autophagy network to provide an intracellular niche for *S. aureus* in human PMNs, but whether it is α-toxin has yet to be determined. The normal autophagic flux, expression of LC3II and p62, was disrupted in PMNs containing *S. aureus* (Mulcahy et al., 2020). *Agr*-positive *S. aureus* leads to the accumulation of autophagy inducer p53 in PMNs, driving transcriptional activation of pro-autophagic membrane protein damage-regulated autophagy monitor (DRAM). DRAM can directly mediate p53-induced autophagy and enhance the accumulation of autophagosomes in cells in order to maintain a survival niche for *S. aureus*. Within these induced autophagosomes, *S. aureus* are protected and ultimately facilitates dissemination. *S. aureus* survival rate is significantly reduced using an *agr*-deficient mutant, suggesting that the *agr* locus is crucial for autophagy-mediated intracellular survival (Mulcahy et al., 2020). Similarly, the *agr* mutant showed a significantly reduced intracellular survival rate in mouse phagocytes because they fail to accumulate LC3-II^+^ autophagosomes and are delivered efficiently to lysosomes (O'Keeffe et al., 2015). These results indicate that *agr*-regulated factors determined the ability of *S. aureus* for autophagy targeting and avoidance of lysosomal degradation in host cells (**Figure 2**). However, in human osteosarcoma cells, *agr*-positive *S. aureus* strains were more efficiently entrapped in autophagosomes than *agr*-negative *S. aureus* (Mauthe et al., 2012). Additionally, a recently study showed the absence of *agr* regulated PSMs increased *S. aureus* long-term survival in human endothelial cells (Siegmund et al., 2021). Thus, a comprehensive analysis of different *S. aureus* strains as well as various cell types is required to elucidate the interplay between *agr* and autophagy.

## Summary and Prospect

The emergence of antibiotic-resistant strains of bacteria requires the continuous development of new antibiotics. However, drug development is a very long and expensive process. The exploration of new drugs for effective treatment of *S. aureus* infection is a difficult task. Many years of research have produced a few drugs, such as penicillin, vancomycin, and cephalosporin. However, once the pathogen becomes intracellular, antibiotics will not achieve the expected effect, and new antibiotics may have an impact on cell function. In this case, development of new molecules must be explored in order to defeat *S. aureus*. Autophagy, as an innate immune response mechanism, degrades *S. aureus* in cells. Strains with high *agr* activity are usually able to escape and replicate intracellularly using autophagy, while strains lacking *agr* systems are usually unable to escape the autophagosomes and are eventually degraded.

Appropriate doses of the autophagy modulators could be an effective strategy for controlling infection. A recent study has reported the natural coumarin derivative daphnetin (DAPH) effectively enhances autophagic pathway to exert an anti-bacterial effect against *S. aureus* (Zhang et al., 2019). Moreover, selenium has been shown to inhibit the proliferation of *S. aureus* by promoting autophagy pathway in *S. aureus* infected mouse macrophages (Zang et al., 2020). Regardless of *S. aureus* strain identity, their methods of escaping autophagy pathway usually involve blocking autophagy flux. The recently developed pH-responsive polymersome (Xu et al., 2020) loaded with LC3 and p62, disintegrates after encountering lysosomes with low pH, releasing the loaded proteins to supplement autophagy flux, which could be a new strategy. However, the situation is more complicated than expected, the fact that autophagy inducers seem to be beneficial for treating *S. aureus* infections, but in turn might facilitate other bacterial infections (Escoll et al., 2016). Therefore, the use of autophagy modulators should be highly cautious. Additionally, the ability of *S. aureus* to escape and survive in the cytosol are dependent on both the strain and cell type. Treatment with autophagy inhibitors was shown to reduce *S. aureus* load, and the autophagy induction by rapamycin restored replication of *S. aureus* (Schnaith et al., 2007; Bravo-Santano et al., 2018). It is difficult to perform corresponding treatment of *S. aureus* infections with different genetic backgrounds and different targeted specific cell types.

We need novel approaches to suppress intracellular *S. aureus* load with minimal side effect to the host. And obviously, the significance of eliminating intracellular bacteria for effective treatment of persistent *S. aureus* infections has received more attention. The vancomycin encapsulated within liposomes was shown to be taken up efficiently by Kupffer cells and killed intracellular *S. aureus*, which reduced the mortality of mice, whereas free vancomycin could not (Surewaard et al., 2016). Combining autophagy modulators with the liposomes may be a promising strategy. The recent focus on developing strategies for intracellular *S. aureus* is encouraging and may lead to more effective treatments in the near future.

## Author Contributions

MW performed the literature survey and wrote the draft. ZF critically reviewed and improved the manuscript. HH contributed to critical evaluation and finalizing of the review. All authors contributed to the article and approved the submitted version.

## Funding

This study was supported by the National Key R&D Program of Intergovernmental Key Projects in China (2018YFE0101700).

## Conflict of Interest

The authors declare that the research was conducted in the absence of any commercial or financial relationships that could be construed as a potential conflict of interest.

## Publisher’s Note

All claims expressed in this article are solely those of the authors and do not necessarily represent those of their affiliated organizations, or those of the publisher, the editors and the reviewers. Any product that may be evaluated in this article, or claim that may be made by its manufacturer, is not guaranteed or endorsed by the publisher.
